# The impact of COVID-19 on psychiatric and mental health services in Europe: suffering experienced by professionals

**DOI:** 10.1186/s12913-022-08776-8

**Published:** 2022-11-16

**Authors:** Hélène Kane, Jade Gourret Baumgart, Emmanuel Rusch, Jocelyn Deloyer, Claudio Fuenzalida, Gabriela Kelemen, Marek Krzystanek, Donatella Marazziti, Margarita Moraitou, Merja Reunanen, Rexhaj Shyhrete, Johannes Thome, Wim Verwaest, Laurence Fond-Harmant, Frédéric Denis

**Affiliations:** 1 EA 75-05 Laboratory of Education, Ethics, Health, Faculty of Medicine, François Rabelais University, Boulevard Tonnellé, Tours, France; 2 St-Martin Neuro Psychiatric Center, Namur, Belgium; 3 Intrafamily Therapy Center, Elche, Spain; 4 Faculty of Education, Psychology and Social Sciences, Aurel Vlaicu University, Arad, Romania; 5 Clinic of Psychiatric Rehabilitation, Faculty of Medical Sciences, Medical University of Silesia, Katowice, Poland; 6 Department of Clinical and Experimental Medicine, University of Pisa, Pisa, Italy; 7 Saint Camillus International, UniCamillus, University of Health Sciences and Medicine, Rome, Italy; 8 Social and Educational Support Center, Kepsipi, Korydallos Greece; 9 Step-Education, Pieksämäki, Finland; 10 School of Nursing Sciences, HES-SO University of Applied Sciences Western Switzerland, Lausanne, Switzerland; 11 Psychiatry Department, Rostock University, Rostock, Germany; 12 Neuro-Psychiatric Hospital Center of Luxembourg, Ettelbruck, Luxembourg; 13 Agency for Europe-Africa Scientific Cooperation, Luxembourg, Luxembourg; 14 EA 3412 Education and Healthcare Practices Laboratories, Sorbonne Paris Nord University, Paris, France

**Keywords:** SARS-CoV-2, COVID-19, Epidemic, Pandemic, Health crisis, Mental health, Psychiatry, Professional practices, Telepsychiatry, Work-life balance, Occupational health, Ethics

## Abstract

**Background:**

The COVID-19 pandemic has not only impacted intensive care units, but all healthcare services generally. This PsyGipo2C project specifically investigates how psychiatry and mental health professionals have been affected by the reorganizations and constraints imposed, which have reshaped their often already difficult working conditions.

**Methods:**

Our research combined quantitative and qualitative methods, surveying and interviewing health professionals of all occupations working in psychiatric and mental health services. A questionnaire was completed by 1241 professionals from 10 European countries, and 13 group interviews were conducted across 5 countries. In addition to this, 31 individual interviews were conducted in Belgium and France.

**Results:**

Among the questionnaire respondents, 70.2% felt that their workload had increased, particularly due to their tasks being diversified and due to increased complexity in the provision of care. 48.9% felt that finding a work-life balance had become more difficult, and 59.5% felt their health had been affected by the crisis. The impact of the health crisis nevertheless varied across professions: our data provides insight into how the health measures have had a differential impact on professional tasks and roles across the various categories of occupations, obliging professionals to make various adaptations. The distress incurred has been linked not only to these new constraints in their work, but also to the combination of these with other pressures in their personal lives, which has consequently compromised their well-being and their ability to cope with multiple demands.

**Discussion:**

The COVID-19 health crisis has had varying impacts depending on the profession and access to remote work, sometimes leading to conflicts within the teams. The suffering expressed by the professionals was tied to their values and patterns of investment in work. Our research also highlights how these professionals made little use of the psychological supports offered, probably due to a reluctance to acknowledge that their mental health was affected.

## Background

Faced with the unprecedented context of the COVID-19 health crisis, the social sciences have provided numerous insights, notably by analyzing how the different States have managed the pandemic from a political perspective [1], and by drawing comparisons with past epidemics [2]. In European countries, the emergence of the COVID-19 pandemic has led to health and organizational crises, with authorities having to create new health systems, sometimes because they were unable to roll out pre-established plans [3]. This crisis revealed disparities in the level of preparedness across national health systems [4], and exacerbated difficulties in health services, some of which were already under stress due to limited human resources. Intensive care units have been placed in the spotlight by the media and in political discourse, as has the heroism of caregivers committed to caring for patients suffering from COVID-19. These front-line professionals undoubtedly incurred a heavy burden in the face of the crisis [5, 6]. Indeed, all health services were disrupted, forced to reorganize in order to prevent the risk of infectious outbreaks, and in some cases scheduled activities had to be cancelled to streamline care.

Psychiatry and mental health services are among those to have been strongly impacted by this health crisis [7, 8], much like intensive care services, which are under severe strain. In these services, reorganizations have been rolled out against the background of pre-existing organizational difficulties within psychiatric and mental health services [9–11]. Moreover, psychiatric services were particularly impacted by physical distancing measures due to the relational nature of their care work [12, 13]. Adopting barrier measures has also been particularly problematic for some patients with psychiatric illnesses [14].

Working conditions in these services had already been difficult for years in many European countries, particularly in relation to the shortage of qualified human resources. Beyond the twofold psychological and physical hardship which is inherent to mental health work [12], there has been a decided lack of resources, hindering the quality of care provided. Indeed, these difficult working conditions are discouraging young professionals from training and working in the mental health sector [15]. These difficulties are all the more worrying as the need for mental healthcare resources has increased in Europe, but also in many countries throughout the world where the health crisis has aggravated the economic difficulties of the most disadvantaged populations [16, 17].

For all of these reasons, professionals in psychiatric services have been severely tested by the COVID-19 pandemic, with the challenges specific to their work [12] being further compounded by new constraints. However, their plight has received little attention, the focus instead being on the burnout of front-line professionals. Healthcare professionals in general are, however, a group which is particularly exposed to the risk of mental health impairment [18, 19]: they have an increased risk of anxiety, depression, burnout, post-traumatic stress, addiction, and suicide [20, 21]. Due to the precarious situation in which psychiatric and mental health services have been operating for years, these risks are heightened among professionals working in these services, compared to those in other disciplines [21].

This article therefore aims to analyze how professionals working in psychiatry and mental health services have been affected by the pandemic. It examines how different categories of professionals experienced the reorganizations and constraints imposed on them during the pandemic, the consequences of these changes on their work-life balance, and their perception of their own mental health in the context of the crisis. These research questions aimed to gain a better understanding of the impact of the pandemic on professionals in order to optimize their health at work.

## Methods

### Study design

In order to study the impact of the COVID-19 health crisis on psychiatry and mental health professionals in Europe, the Psy-GIPO2C project implemented a specific research protocol [22], relied on a consortium of medical and social science researchers, associated with the project as partners and experts, all involved in the various phases of the research. Establishing this international consortium made it possible to distribute the survey to 10 European countries, and to combine different perspectives for the analysis. The study included professionals of all occupations working in psychiatry and mental health services, in order to highlight the variations of perspectives according to professional roles and cultures. The methodology used was a mixed methods approach [23], combining qualitative investigations and in-depth interviews with quantitative investigations with the distribution of an online questionnaire. The qualitative survey aimed to understand the personal experiences of professionals faced with specific situations in their services, while the quantitative survey aimed to produce data to identify and quantify the difficulties experienced by the different categories of professionals.

### Literature review

A systematic review of the international literature [9, 10] was performed according to PRISMA guidelines [24]. The search equations developed enabled us to search for titles and abstracts in PubMed, Cairn, and SantéPsy databases. Articles published in 2020, in English or French, dealing with adjustments put into practice in response to the COVID-19 pandemic by psychiatric and mental health services in several countries around the world were selected. This analysis of the literature led us to develop interview grids for the qualitative survey and a questionnaire framework for the quantitative survey, structured around three common themes: organizational adjustments, use of digital devices, and professional and personal experience of the health crisis.

### Qualitative interviews

Individual and group interviews were conducted between March and May 2021 among professionals practicing in psychiatric departments. The group interviews were conducted in Germany, Belgium, France, Italy, and Luxembourg by our partners in these countries. Individual interviews were furthermore carried out in Belgium and France with volunteer respondents, recruited from several departments to obtain varied feedback. The combination of individual and group interviews enabled variations in the scale of the analysis, also allowing us to document collective experiences as well as individual personal situations. The group interviews promoted the confrontation of different points of view in order to reveal the points of divergence and consensus around shared experiences. Due to the health context, all group interviews were conducted remotely. In contrast, most of the individual interviews were conducted face-to-face, while strictly observing safety measures. Interviews were conducted until the data saturation threshold was reached [25].

All interviews were transcribed in full. The German and Italian transcripts were translated into French. The NVivo© analytical software program (QSR International, Doncaster, Australia) was used to analyze the interviews using a thematic tree. After the tree was validated by all partners and the coding was checked for concordance, two researchers then coded the entire set of interviews. The qualitative analyses were presented to the research consortium, but also reported back to the participants, which allowed the analyses to be critically reviewed and adjusted.

### Questionnaire

The questionnaire, consisting of 59 questions—43 closed and 16 open questions—was developed and edited using Sphinx©. After testing and validating the final version of the questionnaire in the language of their country of practice, the members of the research consortium shared the online questionnaire link between July and November 2021. They shared this link via their professional and institutional social networks (snowball sampling). Professional associations in the field of psychiatry and mental health, or associations involved in activities related to these themes, were also asked to distribute the questionnaire.

Using the analysis tools provided by the Sphinx© software, flat sort and cross sort tables were extracted, and textual analyses of the responses to the open-ended questions were performed.

The qualitative and quantitative data were then analyzed jointly in an iterative process, i.e., through progressive adjustments between the research questions and the data collected [26], remaining mindful of the possible biases inherent to each method. The members of the research consortium came together in September 2021 in Paris for a collective working session to interpret the qualitative and quantitative data collected, and to collectively discuss these analyses.

## Results

### Psychiatry and mental health professionals surveyed

A total of 13 group interviews (GI) and 31 individual interviews (II) were conducted with 96 professionals, both managerial and non-managerial, from health, psychosocial, and administrative services (Table 1). The questionnaire (Q) was completed by 1264 professionals. Countries with fewer than 25 respondents were excluded. Thus, 10 countries and 1241 respondents were included in the analysis (Table 2).

**Table 1:**
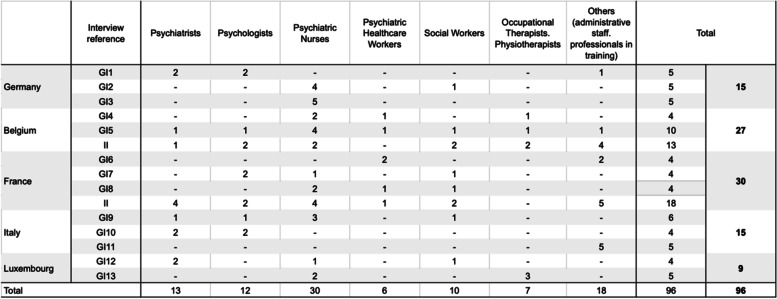
Distribution by occupation of interviewees

**Table 2:**
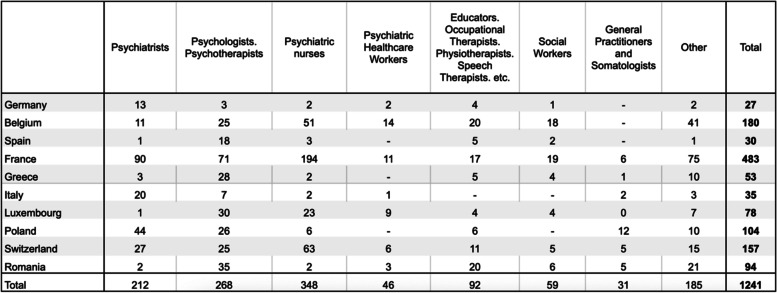
Distribution by occupation of questionnaire respondents

The cross-analysis of both qualitative and quantitative data highlighted the changing demands and constraints these professionals have faced, their difficulties in balancing their professional and personal lives, and the impact of the crisis on their own mental health.

### New professional demands and constraints

The onset of the pandemic has had a considerable impact on the running of psychiatric and mental health services. A number of measures have been implemented within these services: use of individual protective equipment, regular disinfection and ventilation of equipment and premises, carrying out screening tests, dividing the services into smaller units, and creating dedicated quarantine areas. In order to limit contamination, certain activities were reduced: often a reduction in the number of in-patients, suspended out-patient consultations, group and outdoor activities, as well as outings and visits. A significant proportion of the professionals who responded to the questionnaire (25.5%) were obliged to temporarily suspend their work at the beginning of the pandemic. Remote care via digital tools was encouraged (telepsychiatry), as well as working from home (telework). Teams were frequently reorganized, with some professionals assigned to new roles or even to new services.

As a result of these reorganizations, 70.2% of the questionnaire respondents felt their workload had increased overall during the pandemic (Table 3). Analyses of the interviews revealed that the workload fluctuated over the months, with a general decrease in activity in the first few weeks, followed by several successive waves of reorganizations, obliging practitioners to readjust. The constantly changing sanitary protocols have been challenging for them. Absenteeism due to either testing positive for COVID-19 or being contact cases often resulted in a shortage of staff, meaning a heavy workload for those present. The occurrence of COVID-19 outbreaks was also exhausting for those working in the services concerned.

“All our patients were ill, some were less impacted than others. We were swimming in work. It was non-stop, one patient after another.” (Female, Occupational Therapist, Belgium, EI)

**Table 3:**
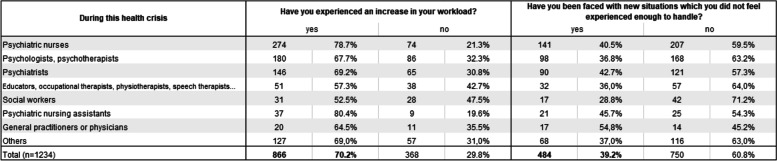
Workload and being faced with new situations, by occupation

However, during certain phases of the health crisis, some of these practitioners paradoxically suffered from an underload of work due, in particular, to therapeutic activities being cancelled. They then experienced feelings of uselessness and powerlessness, the sense that they were unable to fulfil their professional roles.

“We were often blocked. It was a real shame, it was also frustrating for the team... because all ideas we came up with to improve things were blocked at that time.” (Male, Educator, Luxembourg, EG13)

Work overload was more prevalent among nursing assistants and nurses: 80.4% and 78.7% of these, respectively, reported an increase in their workload. As explained in the interviews, this overload was notably linked to the new sanitary, monitoring and screening tasks related to COVID-19. These additional tasks took on considerable importance and engendered increased time commitments. The increase in workload was less frequently reported among social workers (52.5%), and educators, occupational therapists, physiotherapists and speech therapists (57.3%), whose activities were more often suspended. However, these professionals sometimes assisted nursing assistants and nurses.

Furthermore, a significant proportion of questionnaire respondents (39.2%) stated that they had been faced with a situation which they did not feel sufficiently experienced to handle. This was more common among psychiatrists (42.7%), and among physicians working in psychiatry (54.8%), who had to deal with patients suffering from COVID-19, in services with inadequate facilities for this type of care. They also had to deal with new patient profiles whose psychological disorders were triggered or aggravated by the lockdown measures, such as suicidal crises in adolescents and young people. Caregivers also experienced difficult situations (45.7%), particularly due to the complexity of disinfection protocols.

During the interviews, respondents also shared various concerns about the deterioration in the quality of care provided, due to certain therapeutic activities being suspended, restrictions on the freedoms of hospitalized patients, and therapeutic difficulties encountered when monitoring patients remotely.

### Increasing difficulties in balancing professional and personal life

Work-life balance was affected by changes in working hours, increased overtime, on-call duty and increased fatigue due to work intensification. During the pandemic, 48.9% of respondents felt that balancing their family and work life was more difficult (Table 4). Caregivers suffered the most from the situation: 62.5% of nursing assistants and 60.9% of nurses reported that this balance had become more difficult.

**Table 4:**
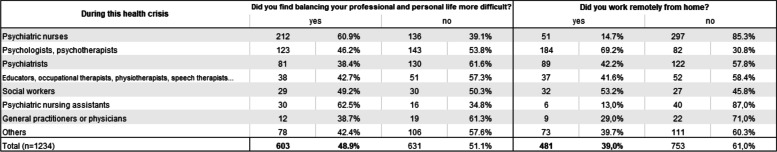
Remote work and work-life balance

The qualitative data showed how difficulties in finding a good work-life balance did not simply result from the impact of work on family life, but rather from an accumulation of work and family difficulties. New constraints on personal life, such as restrictions on leisure and social life, school closures or the impact of the health crisis on family members, certainly contributed to destabilizing some professionals. Given the high mental demands of their work, professionals reported that the restrictions made on the general population had impacted their ability to relax and unwind.

“There was no outlet for venting the accumulated stress. Limited opportunities for recreation heightened this feeling of unease” (Male, Psychologist, Italy, Q)

The work-life balance previously established by professionals have also been challenged by remote work. 39.0% of questionnaire respondents worked remotely from home (Table 4). Access to remote work was very varied across occupations, ranging from 13% of nursing assistants and 14.7% of nurses, to 69.2% of psychologists and psychotherapists. Remote work enabled those professionals who could do so to pursue their work while remaining close to their loved ones without the risk of contaminating them, in contrast to those who had to manage the risk and fear of COVID-19.

“I haven’t been able to see my family, which has been very difficult. My personal life shut down as no one wanted to see me.” (Female, Nurse, Poland, Q)

However, remote working has disrupted the usual work-life balance, particularly due to the blurring of temporal and spatial boundaries. The experience of remote working varied according to family circumstances, especially the presence or absence of children at home, but also according to material and living conditions which made it easier or harder to access information while safeguarding professional secrecy.

“The fact that schools are closed, the difficulty of having to be with your children and work at the same time, the stress, the feeling of not being able to cope and not being 100% there either for your children or for work, not being able to separate spaces and time has generated very intense and frustrating situations.” (Female, Social Worker, Spain, Q)

For some, remote working, combined with an increased demand for availability, has been experienced as an invasion of their personal and family lives. Others spoke of the stress of not leaving home and being with their families constantly.

### Mental health impacted

The surveys highlighted the fact that, added to a general climate marked by restrictions and anxiety, the upheavals in their professional lives have contributed to deteriorated mental health among professionals in psychiatry and mental health services. The responses to the questionnaire provided an idea of the number of professionals concerned: 59.5% consider their mental health to have been affected by this health crisis (Table 5). The proportion of respondents who declared that their mental health had been negatively impacted exceeded 50% for almost all professions, with the exception of general practitioners and physicians. Those who most frequently declared their mental health was affected were nursing assistants (71.7%), nurses (67.8%), social workers (64.4%), psychologists, and psychotherapists (58.6%).

**Table 5:**
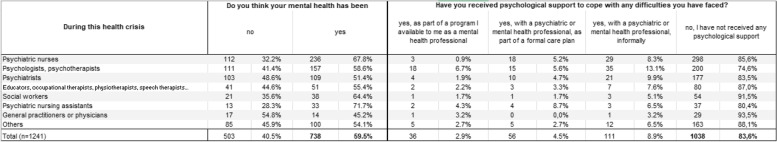
Mental health and seeking psychological support, by occupation

The qualitative data demonstrated the fact that interviewees associated the deterioration of their mental health with new work constraints: fluctuating workloads, stress related to reorganization, fear of being infected or infecting others, the inability to continue some therapeutic activities, and discouragement related to the decline of patients’ health.

“At one point, I had to stop because I felt like crying all the time. That was it, we couldn't take it anymore, the job didn’t make sense to us anymore, we came in to watch over the patients to prevent things from blowing up between them.” (Female, Educator, Belgium, EI)

The healthcare professionals identified signs of mental health impairment in themselves, experiencing difficulties in concentration, anxiety, as well as physical fatigue. In some cases, these difficulties were reflected in a decline in their usual lifestyle, whether in terms of physical activity, diet or tobacco or alcohol consumption.

“During the first lockdown, I drank quite a bit. So that worried me a bit, and I smoked... [...] No, I’m more worried about where we’re going, how we’re going to get there, but... [...] No, it's ok.” (Female, Psychiatrist, France, EI)

Some described how the psychological burden of the health crisis compromised their psychological availability. They sometimes felt saturated and exhausted. Others spoke of being unable to respond to the generalized anxiety caused by the crisis.

“A constant preoccupation with the sense of a threat in the environment, which cannot be adequately dealt with.” (Female, Psychologist, Switzerland, Q)

While 59.5% of respondents reported that their mental health had been affected, 83.6% said they had not sought psychological support (Table 5). 8.9% had received support informally from colleagues, 4.5% had sought help from psychiatric professionals as part of a formal treatment program, and 2.9% had participated in a specific dedicated program. The low uptake of psychological support in the context of formalized care was reported for all professions but was slightly higher among psychologists and psychotherapists (6.7%). The interviews illustrated how the low uptake of formal psychological support, when offered, could have been be due to a reluctance to be the ‘patient’ with colleagues. This enabled us to hypothesize that mental health professionals, reluctant to adopt the status of patient, tended to prefer more informal forms of mutual support. This also indicated that formal support systems must be able to guarantee discretion and anonymity, especially by engaging external and unknown professionals.

## Discussion

Analysis of qualitative and quantitative data from the Psy-GIPO2C survey documented how psychiatric and mental health professionals suffered as a result of the COVID-19 health crisis. This suffering was linked not only to new constraints on their work, but also to other effects of the crisis on their personal and family lives. Three dimensions identified in the results warrant discussion: the differential impact of the crisis according to occupation, the variability of suffering according to ways of working, and the difficulty mental health professionals reported in attending to their own mental health.

### Differentiated impacts of the health crisis, by occupation

Our data have demonstrated how nurses and nursing assistants, who suffered the most from work overload, were also those whose mental health was most affected by the crisis. This is consistent with data from other studies carried out across health services, which have emphasized the particular vulnerability of these professionals [27]. They were exposed to increased hygiene, control, and screening tasks, with this “dirty work” [28] taking on considerable importance and eroding the relational dimension of their work [15]. Hygiene and cleaning activities, which are often overlooked and undervalued, took center stage, in some cases gaining these professionals additional recognition.

The intersection of quantitative and qualitative data has provided insight into how the health measures implemented have transformed the roles and division of labor between different categories of professionals and enabled us to better understand the difficulties encountered by each of these. Although the health crisis has affected the mental health of professionals across all categories of occupation, caregivers have suffered most from an increase in the volume and difficulty of their work, while social workers, educators, occupational therapists and, in some cases, psychologists have suffered more from the impossible task of continuing their activities due to the health measures enforced. Psychiatrists and psychologists more specifically reported difficulties in caring for patients whose disorders worsened as mentioned in the literature [29, 30], and due to a lack of their own psychological availability.

Integrating the various professional profiles, our study has shown how the impact of the pandemic has differed from one occupation to another, but also how it has affected working groups. Indeed, how the professional teams functioned was tested by collective difficulties in reorganizing themselves in response to the constraints, and in coordinating themselves to overcome the restrictions. Moreover, as observed in other fields of activity [31], access to remote work during the COVID-19 pandemic was the source of a reshaping of treatment inequalities between professionals, some suffering due to the obligation to continue their activities in the office, while others suffered from the difficulties of home-based remote work. These inequalities in treatment have sometimes been sources of conflict, whereas team cohesion is important to prevent suffering among professionals in psychiatry [32].

### The extent of distress varied according to the individual's relationship to their work

The COVID-19 health measures have obliged professionals to adapt their professional practices to ensure continuity of care while limiting the risk of viral transmission. These adaptations have left professionals facing contradictory constraints and sometimes ethical dilemmas. Many professionals interviewed expressed how they had suffered from the deterioration in the quality of the care provided, and the deterioration in the health of their patients. Indeed, the literature has demonstrated how reduced care capacities due to COVID-19 may have worsened the condition of people with mental health problems [33]. Dissatisfaction with the quality of the care provided was already very present among psychiatric professionals before the crisis due to insufficient resources [12, 34], but also because their vocation to care for the mentally ill represents an ethic of solidarity [35]. The adjustments made in response to the COVID-19 pandemic have sometimes clashed with professional values, generating suffering in terms of their professional identity [36]. The suffering experienced by mental health professionals during the COVID-19 crisis appeared to be related to the extent of their investment, and to the importance they attributed to their work.

Access to remote work disrupted the balance they had hitherto established in order to meet the respective expectations of their professional and family roles [37]. Faced with more complex work-family balances, they suffered both from not performing their professional role (to the extent that they valued it), and from the spillover of their professional life into their personal life. The forms of suffering experienced thus depended on family configurations and on the importance attributed to their work. Some tended to withdraw into their work, which was the only social space now possible, while others suffered from not being able to devote as much time to their professional activity as before. This may be related to forms of work dependency, as identified in other studies on health professionals [38]. Furthermore, the feeling of non-recognition of their investment in their work has likely aggravated certain forms of work-related suffering among the mental health professionals included in this study [39].

### Stigmatization of mental health in psychiatric and mental health professionals

The mental health of mental health professionals tends to be poorly reported and poorly documented. Despite the significant impact of the health crisis on these practitioners, few of them actually use psychological support services when these are offered. The literature suggests that health professionals, despite being exposed to specific risks, tend to show little concern for their own psychological health [21]. In our study, the professionals interviewed expressed their fears of running into colleagues if they were to use the psychological support systems offered to them. Although they may recognize that their mental health has been affected, they tended to emphasize their ability to overcome these difficulties alone. As psychological resilience is a valued skill, it was difficult for them to acknowledge that they are also vulnerable to mental health disorders [32]. As other studies have shown, psychiatric professionals, as well as the general population, may have a stigmatized view of mental disorders and those affected by them [38]. They also tend to be represented by health professionals in other disciplines as being ‘strange’, with the implication that mental health disorders are contagious and that "you must be crazy yourself to look after crazy people" [40]. We can therefore assume that to counter these representations, psychiatric professionals may be reluctant to acknowledge that their mental health is affected.

Moreover, faced with feelings of discouragement and helplessness linked to the context of the health crisis, we can hypothesize that these professionals will tend to devalue themselves and neglect their own health. However, these psychological resources are essential for them to be able to accompany their patients in their recovery process.

### Limitations

This study, which promotes the complementarity of qualitative and quantitative methods, nevertheless presents certain limitations. The qualitative surveys involved 96 professionals from 5 Western European countries, whereas the quantitative survey was completed by 1241 professionals from 10 countries across Europe. The qualitative surveys present biases related to voluntary recruitment. Specifically, desirability bias likely led interview participants to minimize the impact of the crisis on their personal mental health, with the extent of these difficulties being more apparent in responses to the online questionnaire. For the quantitative surveys, disparities arose in the number of respondents, notably because of the difficulty encountered by partners to mobilize a significant number of professionals in the climate of the health crisis. Seven countries were excluded due to the low number of respondents.

Our study covered a total of 10 European countries but did not take the structural contexts of the countries into account. The situations are therefore difficult to compare given the different contexts of these countries, and due to different public policies and diverse social histories. Nevertheless, given the varied experiences of the professionals surveyed, the Psy-Gipoc2C project shines a spotlight on the transverse nature of the professional cultures and on certain recurring features of psychiatric institutions and the wider mental health field in Europe. The common denominator being the health context of COVID-19: adjustments to professional practices in times of health crisis have transcended borders and contexts.

## Conclusion

Our study shows how the COVID-19 crisis has had a strong impact on the functioning of psychiatric and mental health services, many of which were already in difficulty in Europe, causing suffering at work and impacting the psychological health of the professionals in these services. It is important to preserve the mental health of these professionals, which is often forgotten and stigmatized. To support them, we must promote occupational health in the field of psychiatry and mental health, not only by offering formal psychological support when needed, but also by promoting socialization and mutual support between colleagues. The COVID-19 health crisis has emphasized the importance of shared values forging professional cultures [41], and of the mutual recognition among professionals. In such contexts, it is essential to be able to preserve time-spaces to enable these professionals to interact, and relax and unwind.

## Data Availability

Data are fully available and will be shared upon request to H.K.
